# Potential Role of SESN3 in Linking Heart Failure with Preserved Ejection Fraction and Chronic Obstructive Pulmonary Disease via Autophagy Dysregulation

**DOI:** 10.3390/ijms26115174

**Published:** 2025-05-28

**Authors:** Rongxin Zhu, Binhua Yuan, Yunlin Li, Xiangning Liu, Mingyue Huang, Boyang Jiao, Ying Sun, Sheng Gao, Xiaoqian Sun, Tianhua Liu, Yan Wu, Chun Li

**Affiliations:** 1 School of Traditional Chinese Medicine, Beijing University of Chinese Medicine, Beijing 100029, China; zhurongxin@bucm.edu.cn (R.Z.);; 2 Modern Research Center for Traditional Chinese Medicine, Beijing University of Chinese Medicine, Beijing 100029, China; 3 State Key Laboratory of Traditional Chinese Medicine Syndrome, Guangzhou University of Chinese Medicine, Guangzhou 510006, China; 4 Research Institute of Chinese Medicine, Beijing University of Chinese Medicine, Beijing 100029, China; 5 Key Laboratory of Traditional Chinese Medicine Syndrome and Formula, Ministry of Education, Beijing 100029, China

**Keywords:** HFpEF, COPD, *SESN3*, autophagy, pulmonary dysfunction

## Abstract

Heart failure with preserved ejection fraction (HFpEF) is increasingly recognized as a systemic disorder, often coexisting with chronic obstructive pulmonary disease (COPD). This study aims to identify the shared pathogenic mechanisms between HFpEF and COPD and validate them in an experimental HFpEF model. Transcriptomic datasets from HFpEF cardiac tissue and COPD lung tissue were analyzed using differentially expressed gene (DEG) analysis, weighted gene co-expression network analysis (WGCNA), and functional enrichment analysis. Key genes were identified through least absolute shrinkage and selection operator (LASSO) regression. Immune cell infiltration was assessed using xCell and CIBERSORT, and single-cell RNA sequencing (scRNA-seq) was utilized to determine gene expression patterns across different cell populations. A high-fat diet and N[w]-nitro-L-arginine methyl ester (L-NAME)-induced HFpEF mouse model was established, and the expression of *SESN3* and autophagy-related markers was evaluated in both cardiac and pulmonary tissues using immunofluorescence, quantitative PCR (qPCR), Western blotting (WB), and transmission electron microscopy. DEG and WGCNA analyses identified 1243 and 131 core genes in HFpEF and COPD, respectively. Functional enrichment analysis highlighted autophagy as a common regulatory pathway in both conditions. Among the nine intersecting genes, *SESN3* was identified as a key candidate through LASSO regression. Immune infiltration analysis and scRNA-seq further demonstrated the involvement of *SESN3* in both cardiac and pulmonary pathophysiology. In vivo experiments showed that HFpEF mice exhibited significant lung injury. Furthermore, *SESN3* upregulation and autophagy dysregulation were observed in both heart and lung tissues, supporting a potential systemic role of *SESN3*-mediated autophagy in HFpEF-related pulmonary alterations. This study suggests that *SESN3*-mediated autophagy may represent a shared mechanism between HFpEF and COPD. Our findings suggest that HFpEF may be associated with pulmonary alterations beyond cardiac dysfunction alone. These results provide novel insights into the potential multi-organ involvement in HFpEF and support the role of SESN3 as a shared molecular target in both cardiac and pulmonary pathologies.

## 1. Introduction

Heart failure (HF) is a major clinical syndrome and a leading cause of morbidity and mortality worldwide. Among its subtypes, heart failure with preserved ejection fraction (HFpEF) has now surpassed heart failure with reduced ejection fraction (HFrEF) in prevalence, becoming the dominant form of HF [1]. HFpEF is characterized by a poor prognosis, with five-year survival rates of approximately 35% in hospitalized patients, especially among older individuals with a high comorbidity burden, even lower than that of many cancers [2]. Despite its increasing incidence, the underlying pathophysiological mechanisms of HFpEF remain poorly understood, and effective treatment options are still lacking.

Chronic obstructive pulmonary disease (COPD), the third leading cause of death worldwide, is another major global health challenge due to its high morbidity and economic burden [3,4]. COPD is characterized by persistent airflow limitation and is often accompanied by systemic comorbidities, including cardiovascular diseases, osteoporosis, and metabolic disorders [5]. Notably, approximately one-third of HF patients also have COPD, and the prevalence of COPD is slightly higher in HFpEF patients than in those with HFrEF [6]. Recent studies suggest that COPD is an independent predictor of mortality in HFpEF patients, contributing to both cardiovascular and non-cardiovascular deaths [7,8]. Therefore, COPD and HFpEF may share common pathogenic mechanisms; however, the exact nature of this connection remains unclear.

Emerging evidence indicates that HFpEF is not merely a cardiac disorder but a systemic condition involving widespread metabolic and inflammatory disturbances. While COPD-induced inflammation has been proposed to contribute to HFpEF pathogenesis [9], it remains unclear whether HFpEF itself may also lead to pulmonary alterations through systemic mechanisms. HFpEF is associated with chronic inflammation, oxidative stress, endothelial dysfunction, and extracellular matrix remodeling, all of which could contribute to pulmonary dysfunction [10]. However, the molecular basis linking HFpEF and pulmonary changes remains poorly defined.

**Figure 1:**
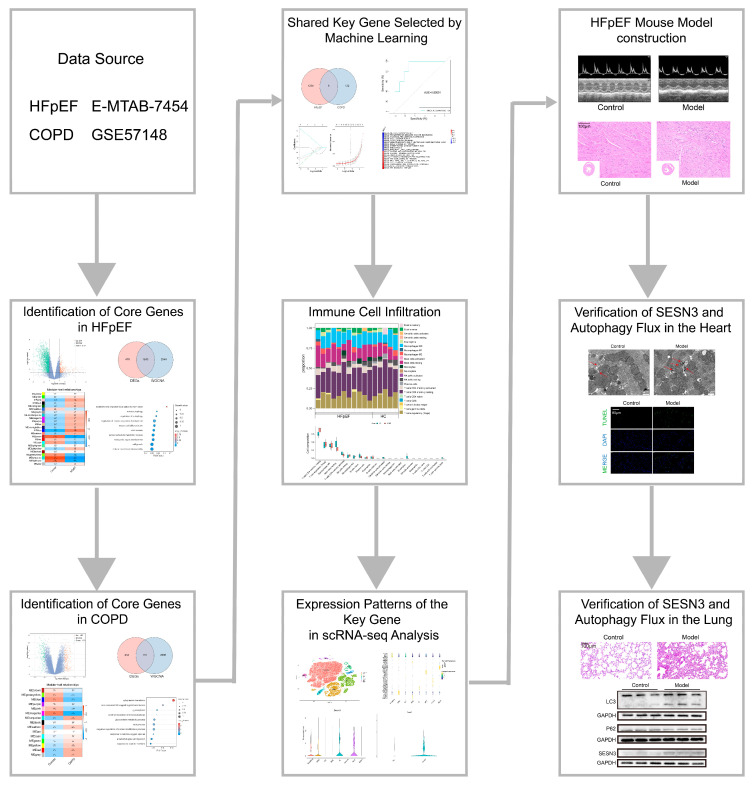
Flow chart of this study.

In this study, we hypothesize that HFpEF and COPD share common pathogenic mechanisms at the molecular level, and that HFpEF may induce pulmonary alterations through these mechanisms. To explore this, we conducted an integrative multi-omics bioinformatics analysis, incorporating weighted gene co-expression network analysis (WGCNA), differentially expressed gene (DEG) analysis, Lasso regression, immune infiltration analysis, and single-cell RNA sequencing (scRNA-seq). We identified *SESN3* as a potential key regulator in both HFpEF and COPD, particularly through its role in autophagy regulation. To further validate our findings, we established an HFpEF mouse model and evaluated *SESN3* expression and autophagy flux in both cardiac and pulmonary tissues using Western blotting (WB), quantitative PCR (qPCR), immunofluorescence, and transmission electron microscopy. Our findings provide new insights into the pathological interplay between HFpEF and COPD, highlighting the potential systemic effects of HFpEF on pulmonary function through shared mechanisms. The flow chart outlining the study design is presented in Figure 1.

## 2. Results

### 2.1. Identification of Core Genes in HFpEF and Functional Enrichment Analysis

**Figure 2:**
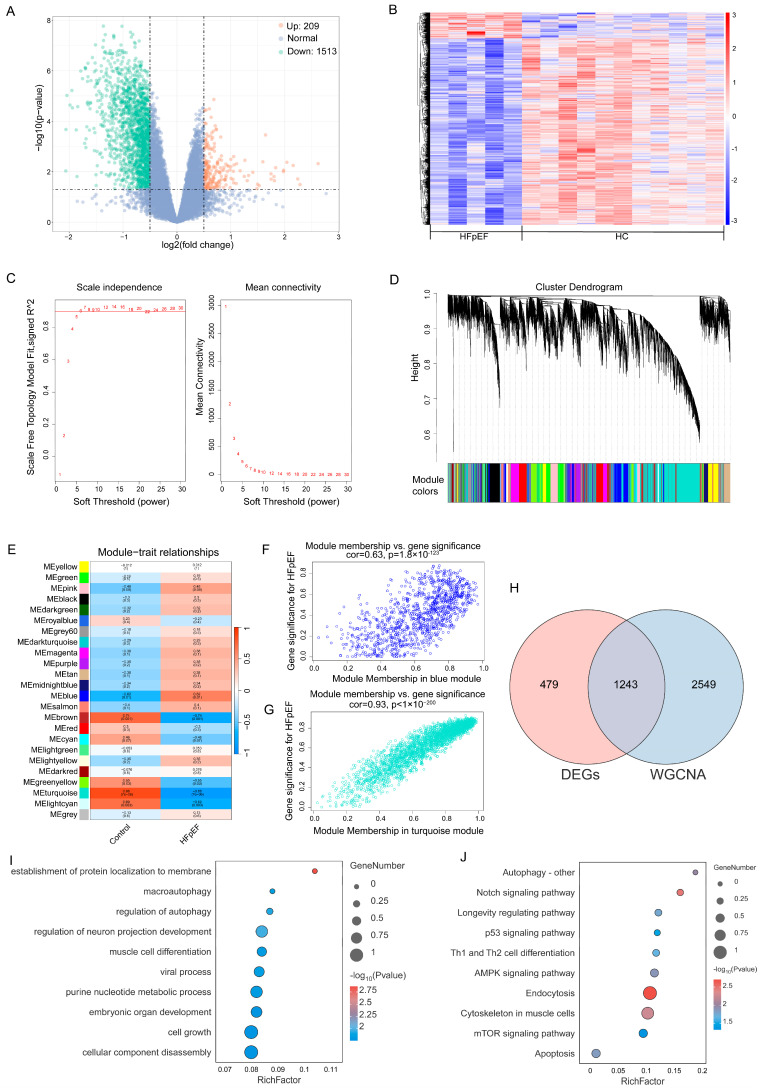
Identification of core genes in HFpEF and functional enrichment analysis. (**A,B**) Volcano plot and heatmap of DEGs in the HFpEF dataset. (**C**) WGCNA soft threshold selection (optimal value = 7) for constructing the network. (**D**) Identification of 24 gene modules. (**E**) Correlation between gene modules and HFpEF. (**F**,**G**) Correlation between gene significance and module membership. (**H**) Venn diagram showing 1,243 overlapping genes between DEGs and WGCNA modules. (**I**) GO enrichment analysis of the overlapping genes. (**J**) KEGG pathway enrichment analysis.

To explore the molecular mechanisms underlying HFpEF, we first performed DEG analysis on the HFpEF dataset E-MTAB-7454, identifying a total of 1722 DEGs (209 upregulated, 1513 downregulated) (Figure 2A,B).

To further identify key HFpEF-associated gene modules, WGCNA was conducted to construct a scale-free network. The optimal soft threshold was determined as seven (Figure 2C), leading to the identification of 24 gene modules with distinct co-expression patterns (Figure 2D). Pearson’s correlation analysis revealed that the blue module exhibited the strongest positive correlation with HFpEF (r = 0.62), while the turquoise module showed a strong negative correlation (r = −0.88) (Figure 2E). Additionally, gene significance (GS) and module membership (MM) were significantly correlated in these key modules, further supporting their association with HFpEF (Figure 2F,G).

By intersecting DEGs from differential expression analysis with WGCNA-identified module genes, we obtained 1243 overlapping genes associated with HFpEF (Figure 2H). To elucidate their potential biological roles, GO and KEGG pathway enrichment analyses were performed.

GO analysis revealed that these genes were significantly enriched in the biological processes related to cell growth, neuronal projection development, muscle cell differentiation, viral processes, purine nucleotide metabolism, protein localization to membranes, and autophagy regulation (Figure 2I).

KEGG pathway analysis further highlighted their involvement in pathways such as endocytosis, apoptosis, the p53 signaling pathway, the longevity-regulating pathway, and autophagy (Figure 2J). Notably, the enrichment of autophagy-related pathways suggests a potential role of autophagic dysregulation in HFpEF pathogenesis, providing a mechanistic link that may extend beyond the heart and impact other organs, such as the lungs.

### 2.2. Identification of Core Genes in COPD and Functional Enrichment Analysis

**Figure 3:**
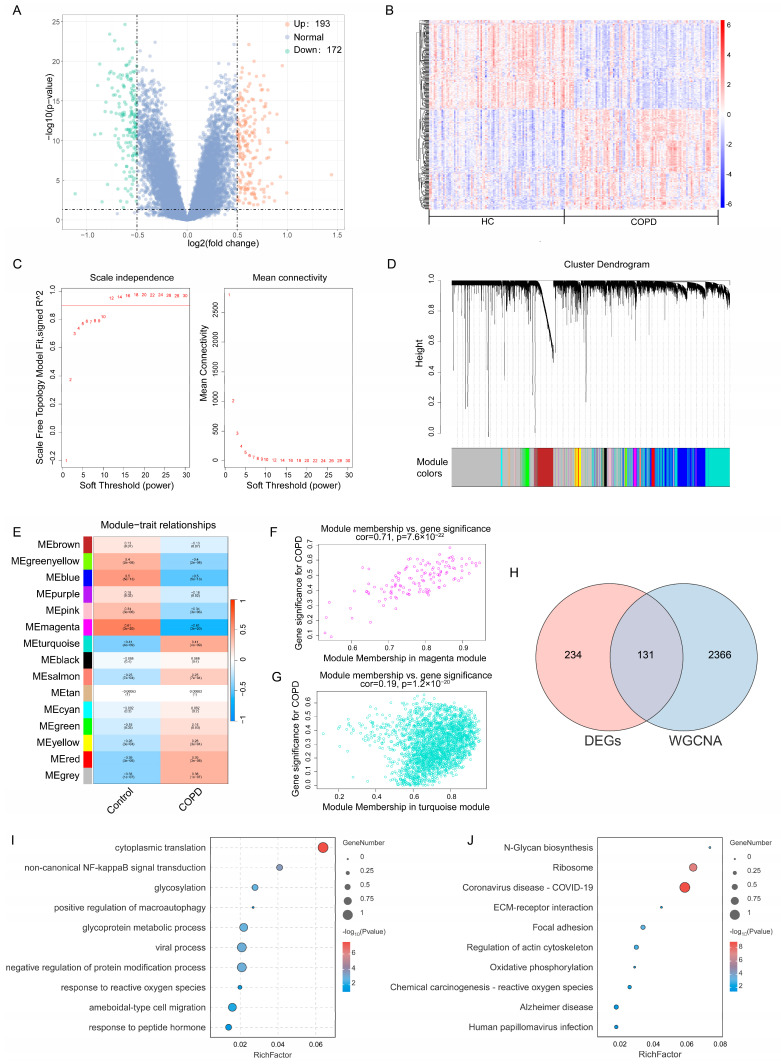
Identification of core genes in COPD and functional enrichment analysis. (**A**,**B**) Volcano plot and heatmap of DEGs in the COPD dataset. (**C**) WGCNA soft threshold selection for network construction. (**D**) Identification of 15 gene modules. (**E**) Correlation analysis between gene modules and COPD. (**F**,**G**) Correlation between gene significance and module membership in key modules. (**H**) Venn diagram showing 131 overlapping genes between DEGs and WGCNA modules. (**I**) GO enrichment analysis. (**J**) KEGG pathway analysis.

To investigate the molecular mechanisms underlying COPD, DEG analysis was conducted on the COPD dataset GSE57148, identifying 365 DEGs, including 193 upregulated and 172 downregulated genes (Figure 3A,B).

Next, WGCNA was performed to construct scale-free networks and identify key gene modules associated with COPD. The soft threshold for GSE57148 was set to 14 (Figure 3C), which led to the identification of 15 distinct gene modules with similar expression patterns (Figure 3D). Pearson’s correlation analysis between module eigengenes and COPD traits revealed that the turquoise module showed a strong positive correlation with COPD (r = 0.41), while the magenta module exhibited a significant negative correlation (r = −0.61) (Figure 3E). Scatter plots further demonstrated a significant correlation between GS and MM, emphasizing their potential role in COPD pathogenesis (Figure 3F,G).

By intersecting the WGCNA-identified disease-related module genes with DEGs from differential expression analysis, we identified 131 overlapping genes associated with COPD (Figure 3H). To explore their biological functions, GO and KEGG enrichment analyses were conducted.

GO analysis revealed that these overlapping genes were significantly enriched in biological processes such as cytoplasmic translation, viral processes, negative regulation of protein modification, response to reactive oxygen species (ROS), and positive regulation of macroautophagy (Figure 3I). The enrichment in autophagy-related pathways is particularly noteworthy, as autophagy dysregulation has been implicated in the pathogenesis of both COPD and HFpEF. This suggests that autophagy could represent a common pathogenic mechanism underlying both diseases.

KEGG pathway analysis revealed that these overlapping genes were involved in pathways such as ribosome function, focal adhesion, N-glycan biosynthesis, and ECM-receptor interactions (Figure 3J). In both the HFpEF and COPD datasets, autophagy-related pathways were significantly enriched. Interestingly, *SESN3* was consistently involved in these pathways across both conditions.

### 2.3. Identification of Shared Key Gene in HFpEF and COPD via Machine Learning

**Figure 4:**
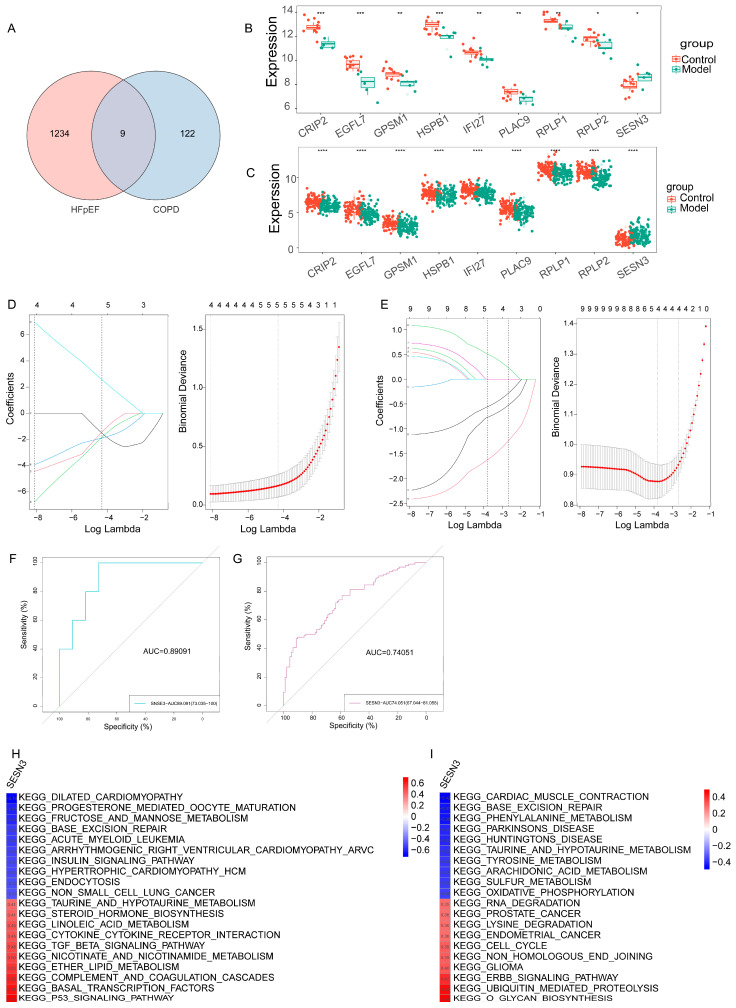
Identification of a shared key gene in HFpEF and COPD via machine learning. (**A**–**C**) Venn diagrams showing nine overlapping genes between HFpEF- and COPD-associated gene sets. (**D**,**E**) LASSO regression analysis identifying key genes in HFpEF and COPD. (**F**,**G**) ROC curve analysis demonstrating the diagnostic potential of *SESN3* in HFpEF and COPD. (**H**,**I**) GSVA heatmaps showing *SESN3*-associated pathways in HFpEF and COPD. The following indicators represent statistical significance: * *p* < 0.05, ** *p* < 0.01, *** *p* < 0.001, **** *p* < 0.0001.

Given the overlap in biological pathways and molecular signatures between HFpEF and COPD, we further sought to identify key shared genes that may serve as potential molecular links between these conditions. A Venn diagram was used to visualize the intersection of overlapping genes identified in HFpEF and COPD, revealing nine shared key genes (Figure 4A–C).

To further prioritize the candidate genes shared by HFpEF and COPD, least absolute shrinkage and selection operator (LASSO) regression was applied, a widely used machine learning technique for high-dimensional data that performs both variable selection and regularization to enhance prediction accuracy. In the HFpEF dataset, four genes—EGFL7, HSPB1, IFI27, and SESN3—were identified as key predictors (Figure 4D). In parallel, the LASSO analysis of the COPD dataset revealed another four key genes—CRIP2, RPLP1, RPLP2, and SESN3 (Figure 4E). Remarkably, SESN3 was the only gene consistently selected across both disease models, suggesting its robust association with the pathophysiology of both HFpEF and COPD. This convergence supports the notion that SESN3 may serve as a potential shared biomarker and therapeutic target in the context of multimorbidity. To assess the diagnostic potential of *SESN3*, we performed receiver operating characteristic (ROC) curve analysis, which demonstrated that *SESN3* effectively distinguished the HFpEF and COPD patients from the healthy controls, with an AUC of 0.89091 in HFpEF and 0.74051 in COPD (Figure 4F,G). These results highlight *SESN3* as a promising diagnostic biomarker for both conditions.

To gain insight into the biological processes influenced by *SESN3*, gene set variation analysis (GSVA) was conducted, revealing its association with key pathways in HFpEF and COPD (Figure 4H,I). The heatmaps illustrate these correlations, with red indicating positive associations and blue indicating negative associations, providing further mechanistic insight into the role of *SESN3* in both diseases.

### 2.4. Immune Cell Infiltration and Its Correlation with Key Genes

Increasing evidence suggests that immune dysregulation plays a crucial role in the pathogenesis of HFpEF and COPD [11,12]. Inflammatory responses and immune cell infiltration contribute to myocardial fibrosis, endothelial dysfunction, and lung parenchymal damage, all of which are hallmarks of these conditions. To investigate the potential immune mechanisms underlying HFpEF and COPD, we employed the xCell and CIBERSORT algorithm to assess immune cell infiltration patterns in both datasets.

**Figure 5:**
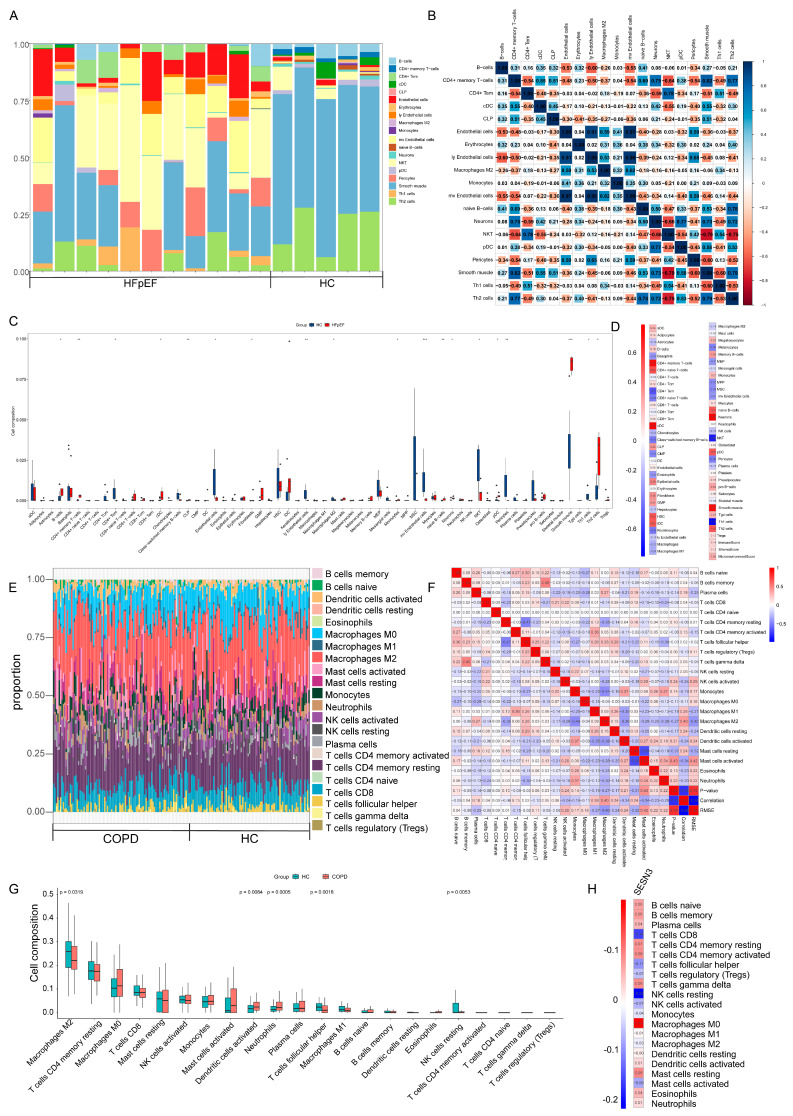
Immune cell infiltration and its correlation with key genes. (**A**,**C**) Comparison of immune cell infiltration between HFpEF patients and healthy controls. (**B**) Heatmap illustrating correlations among immune cell types in HFpEF dataset. (**D**) Correlation analysis between *SESN3* and immune cells. (**E**,**G**) Immune cell infiltration analysis in COPD dataset. (**F**) Correlation of immune cell interactions in COPD dataset. (**H**) Correlation analysis indicating a negative association between *SESN3* and immune cells. The following indicators represent statistical significance: * *p* < 0.05, ** *p* < 0.01, *** *p* < 0.001.

In the HFpEF dataset, significant alterations in B cells, M2 macrophages, NKT cells, Th1 cells, and Th2 cells were observed between the HFpEF patients and the healthy controls (Figure 5A,C). A heatmap was generated to depict correlations among different immune cell types, highlighting their complex interactions (Figure 5B). Correlation analysis revealed a significant negative correlation between SESN3 and regulatory NKT cells (r = −0.66, *p* < 0.05), suggesting a potential immunosuppressive role of SESN3 in HFpEF, possibly contributing to inflammatory resolution and myocardial protection (Figure 5D).

Similarly, in the COPD dataset, significant differences were observed in the infiltration levels of M2 macrophages, dendritic cells, neutrophils, follicular helper T cells, and resting NK cells (Figure 5E,G). The immune correlation heatmap further illustrated the interactions among different immune cell populations (Figure 5F). Notably, *SESN3* exhibited a negative correlation with resting NK cells (r = −0.22, *p* < 0.05), suggesting that *SESN3* may influence innate immune responses and tissue homeostasis in COPD (Figure 5H).

These findings suggest that *SESN3* plays a critical role in immune regulation, differentially influencing immune cell infiltration in HFpEF and COPD. The association of *SESN3* with NKT cells in HFpEF and NK cells in COPD provides novel insights into the intersection of metabolic and immune regulation, further supporting its potential as a therapeutic target.

### 2.5. Expression Patterns of the Key Gene in scRNA-Seq Analysis

To gain deeper insights into the cellular context of *SESN3* expression in HFpEF and COPD, we performed scRNA-seq analysis. This approach enables the identification of cell-type-specific expression patterns, shedding light on the potential functional roles of *SESN3* in disease progression.

**Figure 6:**
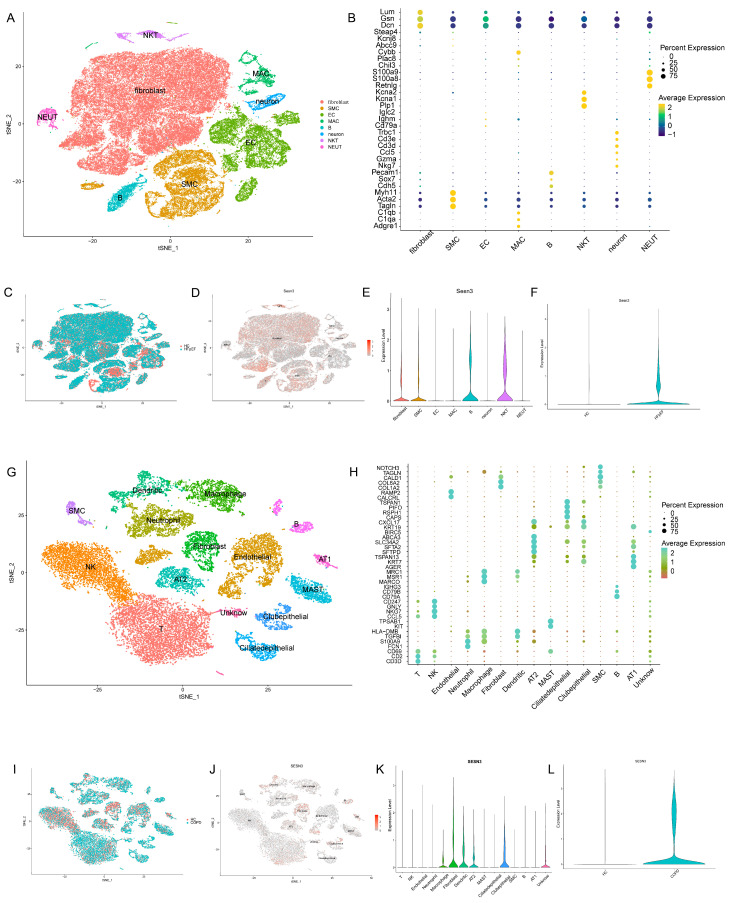
ScRNA-seq analysis of *SESN3* expression in HFpEF and COPD. (**A**,**B**) UMAP plot and cell type annotation in the HFpEF dataset. (**C**) UMAP plot showing *SESN3* expression distribution in HFpEF. (**D**,**E**) Feature plot and violin plot of *SESN3* expression across different cell types in HFpEF. (**F**) Comparison of *SESN3* expression levels between HFpEF and control groups. (**G**,**H**) UMAP plot and cell type annotation in the COPD dataset. (**I**) UMAP plot displaying *SESN3* expression distribution in COPD. (**J**,**K**) Feature plot and violin plot of *SESN3* expression across different cell types in COPD. (**L**) Comparison of *SESN3* expression levels between COPD and control groups.

In the HFpEF dataset, eight distinct cell types were identified based on marker gene expression (Figure 6A,B). *SESN3* expression was primarily detected in fibroblasts, smooth muscle cells (SMCs), B cells, and NKT cells (Figure 6D,E), with a significant upregulation in the HFpEF group compared to healthy controls (Figure 6F). The high expression of *SESN3* in fibroblasts and SMCs suggests a potential role in cardiac fibrosis and vascular remodeling—key pathological features of HFpEF. Additionally, its expression in B cells and NKT cells may indicate an immunomodulatory function.

Similarly, in the COPD dataset, 15 different cell types were identified (Figure 6G,H). *SESN3* was predominantly expressed in macrophages, fibroblasts, dendritic cells, alveolar type 2 (AT2) cells, ciliated epithelial cells, and an unidentified cell population (Figure 6J,K). *SESN3* expression was significantly elevated in the COPD group compared to the healthy controls (Figure 6L). The enrichment of SESN3 in macrophages and AT2 cells suggests potential involvement in chronic inflammatory responses and impaired alveolar repair, respectively—both key features of COPD pathogenesis. Furthermore, its expression in ciliated epithelial cells may indicate a regulatory role in maintaining epithelial integrity and airway remodeling. These findings indicate that *SESN3* plays cell-type-specific roles in HFpEF and COPD.

### 2.6. Validation of the HFpEF Mouse Model: Cardiac Function and Metabolic Alterations

**Figure 7:**
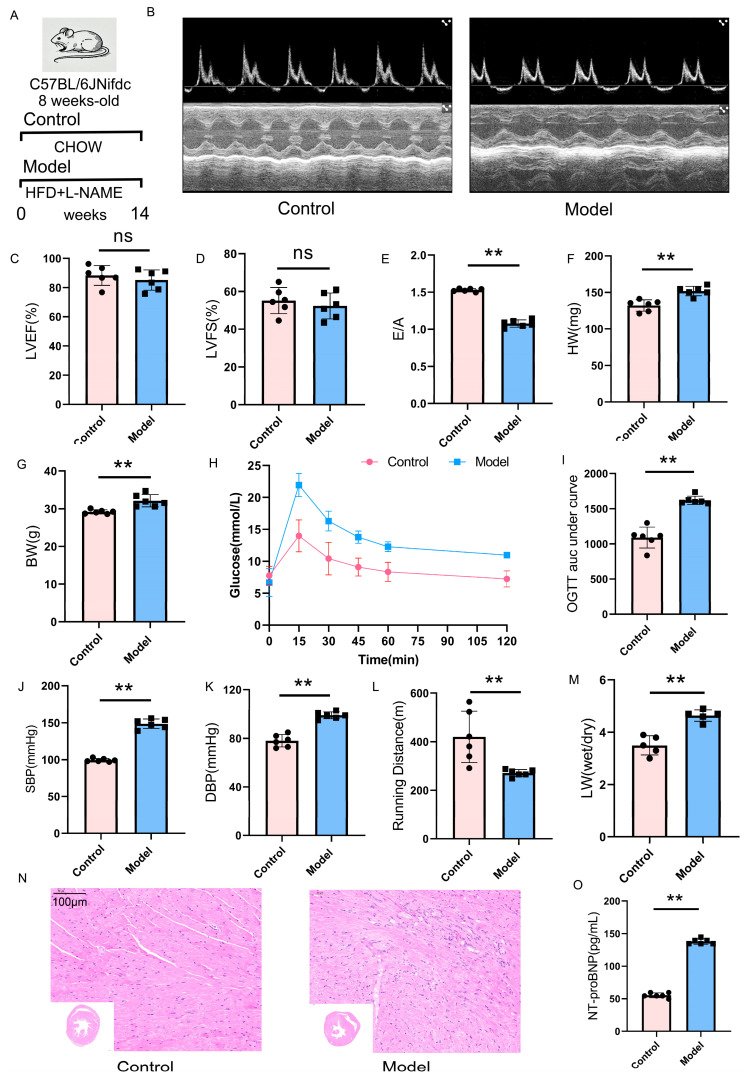
Validation of HFpEF mouse model. (**A**) Schematic diagram of experimental design. (**B**–**D**) Echocardiographic analysis showing preserved LVEF and LVFS. (**E**) Reduced E/A ratio indicating impaired diastolic function. (**F**) Increased left ventricular mass in HFpEF mice. (**G**) Body weight changes over study period. (**H**,**I**) Glucose tolerance test showing impaired glucose metabolism in HFpEF mice. (**J**,**K**) Elevated systolic and diastolic blood pressure following L-NAME administration. (**L**) Treadmill endurance test demonstrating reduced exercise capacity. (**M**) Increased lung wet-to-dry weight ratio, suggesting pulmonary congestion. (**N**) HE staining of mouse heart. (**O**) Serum NT-proBNP levels. Scale bar = 100 μm. The notation “ns” signifies no significant difference, the following indicators represent statistical significance: ** *p* < 0.01.

To establish an HFpEF mouse model, we subjected mice to HFD feeding combined with L-NAME administration for 14 weeks. The experimental design is illustrated in Figure 7A.

Echocardiographic analysis revealed that while LVEF and LVFS remained preserved (Figure 7B–D), HFpEF mice exhibited a significant reduction in the E/A ratio (Figure 7E), indicating impaired diastolic function. Additionally, a substantial increase in left ventricular mass was observed (Figure 7F), further confirming the development of cardiac remodeling characteristic of HFpEF.

Mice subjected to HFD treatment exhibited progressive weight gain (Figure 7G), accompanied by impaired glucose metabolism as evidenced by glucose tolerance testing (Figure 7H,I). Furthermore, L-NAME administration resulted in a significant elevation of both systolic and diastolic blood pressure (Figure 7J,K), consistent with systemic vascular dysfunction observed in HFpEF patients.

To assess exercise capacity and pulmonary congestion, we performed treadmill endurance testing and measured the lung wet-to-dry weight ratio. HFpEF mice demonstrated a marked decline in running distance (Figure 7L), indicative of exercise intolerance, a hallmark symptom of HFpEF. Moreover, the lung wet-to-dry weight ratio was significantly elevated (Figure 7M), suggesting pulmonary congestion and early signs of heart failure-related lung pathology. The hearts of HFpEF mice exhibited a disordered arrangement of cardiac tissue, expanded intercellular spaces, and noticeable cell loss (Figure 7N). The serum NT-proBNP levels were elevated in the HFpEF group (Figure 7O).

Collectively, these findings confirm the successful establishment of an HFpEF mouse model that closely recapitulates key pathophysiological features of the disease, including preserved systolic function, diastolic dysfunction, cardiac hypertrophy, metabolic abnormalities, systemic hypertension, exercise intolerance, and pulmonary congestion.

### 2.7. Verification of SESN3 and Autophagy Flux in the Heart

**Figure 8:**
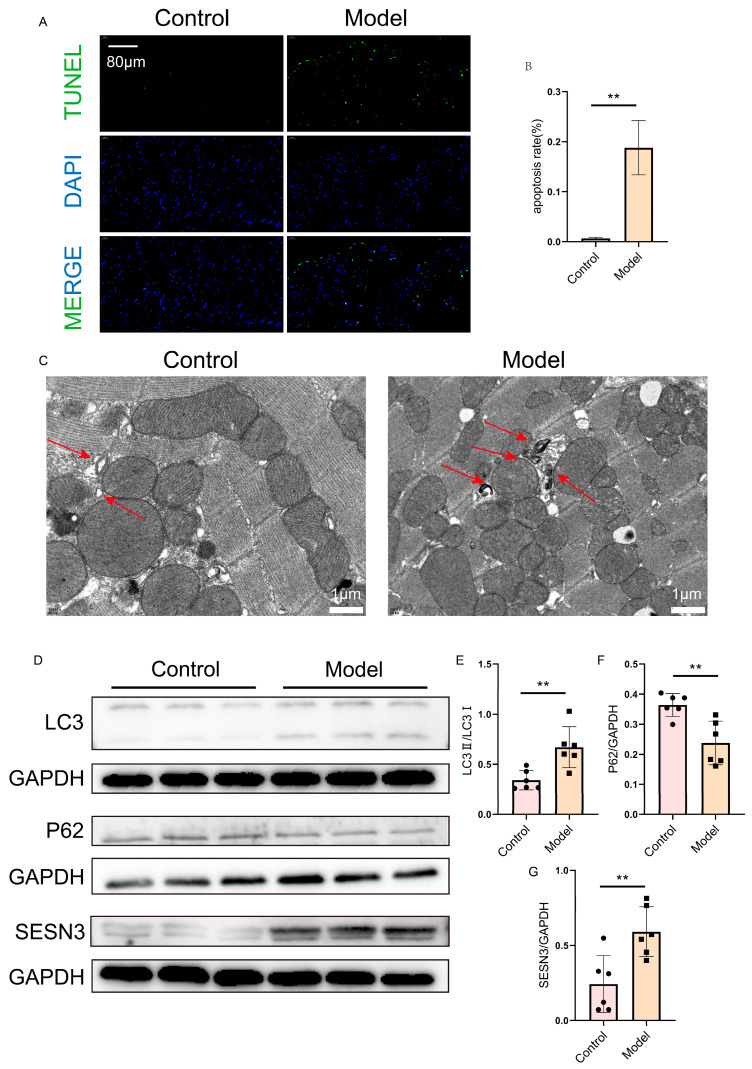
Verification of SESN3 expression and autophagy flux in heart. (**A**,**B**) TUNEL staining showing increased cardiomyocyte apoptosis in HFpEF mice. Scale bar = 80 μm. (**C**) Transmission electron microscopy (TEM) images revealing increased autophagosome accumulation in HFpEF cardiomyocytes. The red arrow points to the autophagosomes. Scale bar = 1 μm. (**D**–**G**) Western blot analysis showing upregulated SESN3 and key autophagy-related proteins in HFpEF heart tissue. The following indicators represent statistical significance: ** *p* < 0.01.

To investigate cardiomyocyte apoptosis in HFpEF mice, TUNEL staining was performed. The results demonstrated a significant increase in apoptotic cardiomyocytes in the HFpEF model group compared to the control group (Figure 8A,B).

To further examine autophagy levels in the hearts of HFpEF mice, transmission electron microscopy (TEM) was utilized. The TEM images revealed a notable accumulation of autophagosomes in cardiomyocytes of the HFpEF model group compared to the control group (Figure 8C).

Most importantly, Western blot analysis showed that the expression levels of SESN3 and key autophagy-related proteins exhibited significant differences in expression in the HFpEF model group compared to the control group (Figure 8D–G). These findings confirm the altered autophagic activity in HFpEF hearts and highlight the pivotal role of SESN3 in both HFpEF pathology and autophagy regulation.

### 2.8. Verification of SESN3 and Autophagy Flux in the Lung

**Figure 9:**
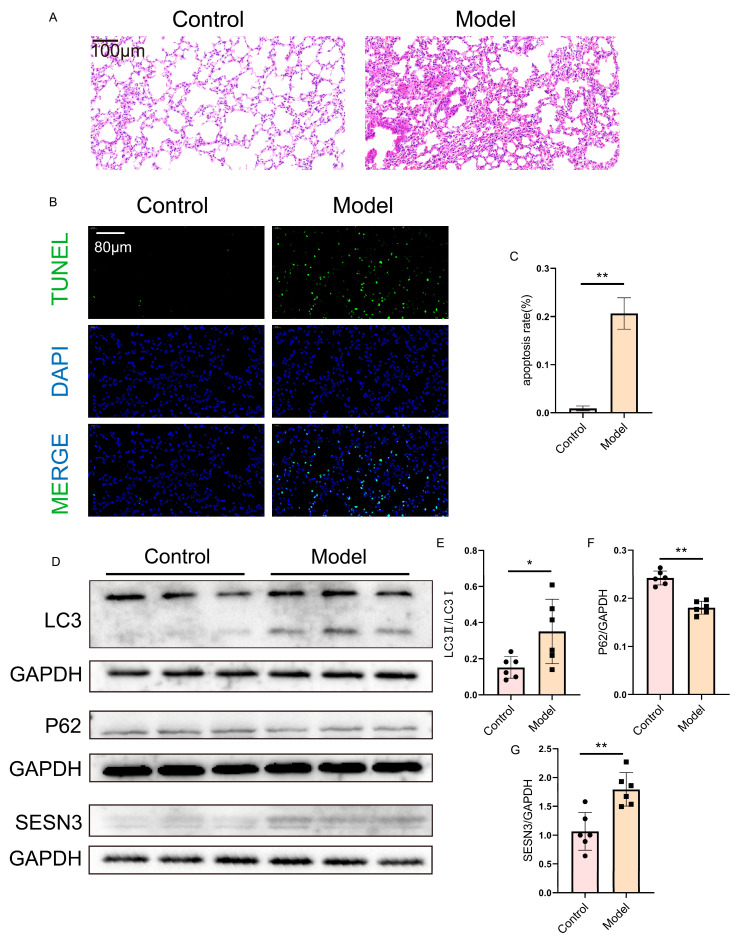
Verification of SESN3 expression and autophagy flux in lung. (**A**) HE staining showing inflammatory infiltration and bronchial lumen narrowing in HFpEF lung tissues. Scale bar = 100 μm. (**B**,**C**) TUNEL staining indicating increased apoptosis in HFpEF lung tissues. Scale bar = 80 μm. (**D**–**G**) Western blot analysis demonstrating upregulated SESN3 and autophagy-related proteins in HFpEF lung tissues. The following indicators represent statistical significance: * *p* < 0.05, ** *p* < 0.01.

HE staining of the lung tissues from the HFpEF mice revealed pronounced inflammatory infiltration and bronchial lumen narrowing in the model group compared to the control group (Figure 9A).

Additionally, TUNEL staining indicated a significant increase in apoptotic cells in the lung tissues of the HFpEF mice relative to the controls (Figure 9B,C).

Furthermore, Western blot analysis revealed altered expression levels of SESN3 and autophagy-related proteins in the lung tissues of HFpEF mice compared to the control group (Figure 9D–G). These findings indicate that SESN3 may be a key regulator of autophagy in the pulmonary pathology of HFpEF.

## 3. Discussion

HFpEF and COPD are both significant global health concerns, each with high morbidity and mortality rates. While HFpEF and COPD are distinct conditions, their overlapping pathophysiological mechanisms—particularly in relation to inflammation, oxidative stress, and autophagy—highlight the potential for shared molecular pathways. A recent analysis showed that COPD was the only comorbidity with a significant interaction (*p* < 0.01) between EF group and outcomes. It posed a higher mortality risk in HFpEF than in HFrEF [13]. An alternative explanation for the COPD–HFpEF link is the interaction between impaired LV filling and pulmonary venous changes from lung parenchymal abnormalities [14]. Moreover, HF may result in pulmonary function changes and patient symptoms that mimic COPD [15]. In this study, we aimed to explore the molecular and cellular mechanisms linking HFpEF and COPD, particularly focusing on *SESN3* as a central regulator in both diseases.

*SESN3* was identified as a key gene through multiple analytical approaches, including differential expression analysis, WGCNA, and LASSO regression models. Notably, *SESN3* was the only gene consistently found in both HFpEF and COPD datasets, suggesting its potential role as a common molecular link between these diseases. Our results also demonstrated that *SESN3* expression was elevated in both the heart and lungs of HFpEF mice, suggesting its involvement in systemic disease processes that extend beyond the heart. This finding underscores the systemic nature of HFpEF, as it involves not only the cardiac pathology but also pulmonary alterations.

The Sestrin family consists of the following three members: *SESN1*, *SESN2*, and *SESN3*. *SESN1* is widely expressed in human tissues, with particularly high levels in skeletal muscle, heart, liver, and brain. *SESN2* has been identified as a hypoxia-inducible gene, while *SESN3* remains the least studied among them [16,17,18]. Sestrins play a crucial role in maintaining metabolic homeostasis, primarily by interacting with the AMPK/mTORC1 signaling pathway to regulate glucose homeostasis, insulin sensitivity, and lipid metabolism. Additionally, they contribute to cellular stress responses by modulating autophagy. Under stress conditions, Sestrins enhance autophagy through their interaction with the AMPK/mTOR signaling axis [19,20,21,22,23].

Although our study does not provide direct causal evidence, previous research supports SESN3 as a functional modulator of autophagy. SESN3 is a known downstream target of FOXO1 and p53, and it regulates autophagy via AMPK–mTOR signaling [24]. In pulmonary hypertension models, SESN3 mediates FOXO1-induced autophagy and suppresses mTOR activity [25]. Additionally, Sestrins mitigate oxidative stress and aging-related damage—key features in both HFpEF and COPD [26]. These findings, along with our results, suggest that SESN3 may contribute to autophagy dysregulation across cardiac and pulmonary tissues.

The identification of autophagy-related pathways in both HFpEF and COPD provides further evidence of their shared pathogenic mechanisms. Autophagy is a critical cellular process that regulates the degradation of damaged organelles and proteins, playing a pivotal role in maintaining cellular homeostasis. Dysregulated autophagy has been implicated in the pathogenesis of various diseases, including cardiovascular and pulmonary diseases [27,28]. In this study, we observed significant alterations in autophagic flux in the hearts and lungs of HFpEF mice, accompanied by increased *SESN3* expression. These findings suggest that *SESN3*-mediated autophagy dysregulation may contribute to the pathology of HFpEF. Importantly, HFpEF is not merely a cardiac disorder but a systemic condition that affects multiple organs, including the lungs, kidneys, and skeletal muscles, through mechanisms such as chronic inflammation, endothelial dysfunction, and metabolic disturbances [29,30,31]. While we did not have access to COPD lung tissues for direct validation, our findings in HFpEF lungs suggest that similar autophagic dysregulation could be present in COPD. Rather than directly inducing COPD, HFpEF may contribute to pulmonary dysfunction as part of its systemic impact, further highlighting the complex interplay between cardiovascular and respiratory diseases.

Immune cell infiltration also appears to play a key role in the pathogenesis of both HFpEF and COPD. Our immune infiltration analysis indicates that *SESN3* is associated with NKT cells in HFpEF, and with natural killer (NK) cells in COPD, suggesting that *SESN3* might regulate immune responses differently in the context of each disease. In HFpEF, the correlation between *SESN3* and NKT cells may suggest a potential immunosuppressive role, contributing to inflammatory resolution and myocardial protection. Conversely, in COPD, *SESN3*’s negative correlation with NK cells may indicate its role in modulating innate immune responses and maintaining tissue homeostasis. These findings suggest that *SESN3* not only regulates autophagy but also influences immune responses in a cell-type-specific manner, further highlighting its potential as a therapeutic target for both diseases.

The experimental validation using the HFpEF mouse model provides critical insights into the pathological features of the disease, confirming the successful establishment of a model that mimics key clinical characteristics of HFpEF, including impaired diastolic function, cardiac hypertrophy, and pulmonary congestion. However, a limitation of our study is the absence of COPD lung tissues for direct validation of the observed autophagic changes in the lungs. While our findings in HFpEF lung tissues provide suggestive evidence of autophagy dysregulation, future studies using COPD animal models are needed to confirm whether similar mechanisms operate in COPD.

In conclusion, our study suggests that SESN3 may serve as a potential molecular link between HFpEF and COPD through its association with autophagy and immune responses. However, further studies are needed to determine whether SESN3 plays a causal or compensatory role in disease progression. Notably, HFpEF is increasingly recognized as a systemic disorder that extends beyond cardiac dysfunction, affecting multiple organs, including the lungs, kidneys, and skeletal muscles, through chronic inflammation, endothelial dysfunction, and metabolic disturbances. Although our findings in HFpEF lungs provide insights into the molecular mechanisms linking HFpEF and COPD, further studies using COPD models are needed to fully elucidate the shared pathogenic mechanisms and validate *SESN3* as a therapeutic target. Understanding HFpEF as a multi-organ syndrome rather than an isolated cardiac condition may open new avenues for therapeutic strategies addressing its systemic effects.

## 4. Materials and Methods

### 4.1. Data Collection and Processing

Gene expression data for HFpEF (E-MTAB-7454) were obtained from the ArrayExpress database, while COPD-related gene expression data (GSE57148) were retrieved from the Gene Expression Omnibus (GEO) database. WGCNA were performed separately to identify overlapping genes in HFpEF and COPD. The intersection of these overlapping genes was designated as shared genes, and core genes were further selected using the least absolute shrinkage and selection operator (LASSO) regression. The CIBERSORT algorithm was employed to estimate immune infiltration levels, while scRNA-seq data were used to validate the expression patterns of core genes. Additionally, an HFpEF mouse model was established to verify the expression levels of key genes and pathways.

### 4.2. Identification of Differentially Expressed Genes (DEGs)

The “limma” package (version 3.60.4) was utilized to identify differentially expressed genes (DEGs). Genes with a |log2(foldchange)| > log2(1.5) and *p* < 0.05 were classified as DEGs. DEGs were visualized using volcano and heat maps generated with the “ggplot2” package (version 3.5.1). The false discovery rate was controlled by adjusting values using the Benjamini–Hochberg algorithm.

### 4.3. Weighted Gene Co-Expression Network Analysis

WGCNA was conducted to identify gene modules with significant co-expression relationships [32]. The “WGCNA” package (version 1.72.5) was used to determine the soft-threshold power (β) via the “PickSoftThreshold” function, selecting the optimal value. Weighted correlation analysis and hierarchical clustering were then performed to identify gene modules, each of which was assigned a unique color representation. Disease-related modules exhibiting significant regulatory fluctuations were further analyzed for correlation. Ultimately, modules significantly associated with disease traits were selected, and their constituent genes were identified as key disease-related genes.

### 4.4. Functional Enrichment Analysis

To explore the molecular functions associated with the target gene sets, gene annotation was performed using the “clusterProfiler” package (version 4.10.0) in R. Functional enrichment analysis was conducted using the “clusterProfiler” package (version 4.10.0) with default parameters, incorporating data from the Gene Ontology (GO) database (https://www.geneontology.org/ (accessed on 20 February 2025)) and the Kyoto Encyclopedia of Genes and Genomes (KEGG) database (https://www.kegg.jp/ (accessed on 20 February 2025)). Enrichment results were visualized using the “ggplot2” package (version 3.4.4) and “GOplot” package (version 1.0.2), with pathways showing *p* < 0.05 considered significantly altered.

### 4.5. Core Gene Selection via LASSO

LASSO regression was applied to identify shared biomarkers between HFpEF and COPD, given its suitability for high-dimensional datasets, such as gene expression profiles. By implementing L1 regularization, LASSO regression effectively performed feature selection, reducing model complexity and mitigating overfitting [33].

### 4.6. Gene Set Variation Analysis (GSVA)

GSVA was conducted using the “GSVA” R package (version 1.50.5) to compute enrichment scores for gene sets across individual samples [34]. Pearson’s correlation analysis was performed to examine the relationships between hub genes and gene sets, with results visualized using the “pheatmap” package (version 1.0.12).

### 4.7. Immune Infiltration and Correlation Analysis

The xCell and CIBERSORT algorithms were employed to assess immune infiltration patterns in HFpEF and COPD datasets, with *p* < 0.05 considered statistically significant. Wilcoxon rank-sum tests were used to compare immune cell distributions between HFpEF and control groups, as well as between COPD and control groups. Pearson’s correlation analysis, visualized using the “pheatmap” package, was performed to assess associations between hub genes and immune cell types.

### 4.8. Single-Cell Data Processing and Gene Expression Analysis

ScRNA-seq datasets from HFpEF mouse models and COPD patients were obtained from GEO (GSE236586 and GSE173896) [35,36]. Data were processed using the “Seurat” package (version 5.2.1), and cells with nFeature > 500 were selected for further analysis. Batch effects across samples were corrected using the “harmony” package (version 1.2.3). Uniform Manifold Approximation and Projection (UMAP) was used for dimensionality reduction and cell clustering. Cells were annotated using validated marker genes, and differentially expressed genes within each cluster were identified using the “FindAllMarkers” function. Visualization of shared biomarkers was performed using the “ggplot2” package (version 3.4.4) and “Seurat” package.

### 4.9. Experimental Animals

Male C57BL/6JNifdc mice (20 ± 2 g, 6–8 weeks old) were obtained from SPF (Beijing) Biotechnology Co., Ltd. (Beijing, China). All animal procedures were conducted following the guidelines of the Institutional Animal Care and Use Committee (IACUC) and approved by the Animal Care Committee of Beijing University of Chinese Medicine (Registration number: BUCM-2024060702-2161). Mice were housed in a pathogen-free facility with ad libitum access to food and water.

The HFpEF model was induced using a two-hit strategy [37]. After a week of acclimation, mice were randomly divided into two groups—control and HFpEF. The HFpEF group was fed a 60% high-fat diet (Beijing SibeiFu Biotechnology Co., Ltd., Beijing, China) along with drinking water containing N[w]-nitro-L-arginine methyl ester (L-NAME; 0.5 g/L, Sigma Aldrich, St. Louis, MO, USA). The control mice received a standard diet and water without L-NAME. At 14 weeks, successful modeling was confirmed by echocardiography, and metabolic and functional evaluations, including oral glucose tolerance tests (OGTTs), blood pressure measurements, and exercise capacity assessments, were performed.

### 4.10. Echocardiographic Assessment

Transthoracic echocardiography was performed using a VisualSonics Vevo 2100 system with an MS400 transducer (Visualsonics, Toronto, ON, Canada). Left ventricular ejection fraction (LVEF) and other systolic function indices were obtained from short-axis M-mode scans. Diastolic function was assessed using pulsed-wave and tissue Doppler imaging in anesthetized mice.

### 4.11. Blood Pressure Measurement

Systolic blood pressure was measured noninvasively using the tail-cuff method (Kent Scientific, Torrington, CT, USA). Mice were placed in individual holders on a temperature-controlled platform (37 °C), and readings were averaged over at least eight measurements per session.

### 4.12. Glucose Tolerance Test

OGTT was conducted by administering orally glucose (2 g/kg) after a 6 h fast. Blood glucose levels were recorded at 0, 15, 30, 45, 60, and 120 min using a glucometer.

### 4.13. Exercise Exhaustion Test

After three days of treadmill acclimation, an exercise exhaustion test was performed. Mice ran uphill (20°) starting at 5 m/min for 4 min, followed by incremental speed increases of 2 m/min every 2 min until exhaustion. Running time and distance were recorded.

### 4.14. Histological Analysis

Heart and lung tissues were fixed in 4% paraformaldehyde for 48 h, embedded in paraffin, and sectioned at 4 μm. Hematoxylin and eosin (HE) staining was performed for histopathological evaluation.

### 4.15. Transmission Electron Microscopy (TEM)

Heart tissues were fixed in 2.5% glutaraldehyde at 4 °C overnight and post-fixed in 1% osmium tetroxide. Dehydrated samples were embedded in resin, sectioned, and stained with uranyl acetate and lead citrate before imaging under a transmission electron microscope (Leica, Buffalo Grove, IL, USA).

### 4.16. Western Blot Analysis

Protein concentrations were determined using a BCA assay. Proteins were separated via SDS-PAGE, transferred onto PVDF membranes, and incubated with primary and secondary antibodies. They were imaged using an imager (Bio-Rad, Hercules, CA, USA) following incubation with an enhanced chemiluminescence detection reagent at room temperature for 1 min. The band densities were analyzed and quantified using ImageJ software (version 1.53K). The antibodies used for WB were as follows: LC3 Recombinant antibody (#81004-1-RR, Proteintech Group, Inc., Rosemont, IL, USA); SQSTM1/p62 Antibody (#AF5384, Affinity Biosciences, Cincinnati, OH, USA); SESN3 Polyclonal antibody (#11431-2-AP, Proteintech Group, Inc.); anti-GAPDH antibody (#ab263962, Abcam, Cambridge, UK); and HRP Anti-Rabbit IgG antibody (#ab270144, Abcam, Cambridge, UK).

### 4.17. Statistical Analysis

Statistical analyses were performed using GraphPad Prism 6.0. Data are presented as mean ± SD. Two-group comparisons were conducted using an unpaired two-tailed t-test, while multiple comparisons were analyzed using ANOVA followed by Bonferroni post hoc tests. *p* < 0.05 was considered statistically significant.

## 5. Conclusions

In this study, we systematically elucidated the molecular mechanisms connecting HFpEF and COPD through integrative bioinformatics analysis and experimental validation. Autophagy was identified as a shared pathogenic pathway in both diseases, with SESN3 acting as a potential key regulator. Upregulated SESN3 and dysregulated autophagy were observed in both cardiac and pulmonary tissues of HFpEF mice, indicating that HFpEF may induce systemic effects contributing to pulmonary dysfunction. These findings provide novel insights into the systemic nature of HFpEF and suggest that the SESN3–autophagy axis may serve as a promising therapeutic target for addressing multi-organ involvement associated with HFpEF.

## Data Availability

The original contributions presented in this study are included in the article. Further inquiries can be directed to the corresponding author(s).
